# Oxygen Glucose Deprivation in Rat Hippocampal Slice Cultures Results in Alterations in Carnitine Homeostasis and Mitochondrial Dysfunction

**DOI:** 10.1371/journal.pone.0040881

**Published:** 2012-09-11

**Authors:** Thomas F. Rau, Qing Lu, Shruti Sharma, Xutong Sun, Gregory Leary, Matthew L. Beckman, Yali Hou, Mark S. Wainwright, Michael Kavanaugh, David J. Poulsen, Stephen M. Black

**Affiliations:** 1 Department of Biomedical and Pharmaceutical Sciences, University of Montana, Missoula, Montana, United States of America; 2 Vascular Biology Center, Medical College of Georgia, Augusta, Georgia, United States of America; 3 Department of Pediatrics, Northwestern University Feinberg School of Medicine, Children's Memorial Hospital, 2300 Children's Plaza, Chicago, Illinois, United States of America; Universidad de Castilla-La Mancha, Spain

## Abstract

Mitochondrial dysfunction characterized by depolarization of mitochondrial membranes and the initiation of mitochondrial-mediated apoptosis are pathological responses to hypoxia-ischemia (HI) in the neonatal brain. Carnitine metabolism directly supports mitochondrial metabolism by shuttling long chain fatty acids across the inner mitochondrial membrane for beta-oxidation. Our previous studies have shown that HI disrupts carnitine homeostasis in neonatal rats and that L-carnitine can be neuroprotective. Thus, this study was undertaken to elucidate the molecular mechanisms by which HI alters carnitine metabolism and to begin to elucidate the mechanism underlying the neuroprotective effect of L-carnitine (LCAR) supplementation. Utilizing neonatal rat hippocampal slice cultures we found that oxygen glucose deprivation (OGD) decreased the levels of free carnitines (FC) and increased the acylcarnitine (AC): FC ratio. These changes in carnitine homeostasis correlated with decreases in the protein levels of carnitine palmitoyl transferase (CPT) 1 and 2. LCAR supplementation prevented the decrease in CPT1 and CPT2, enhanced both FC and the AC∶FC ratio and increased slice culture metabolic viability, the mitochondrial membrane potential prior to OGD and prevented the subsequent loss of neurons during later stages of reperfusion through a reduction in apoptotic cell death. Finally, we found that LCAR supplementation preserved the structural integrity and synaptic transmission within the hippocampus after OGD. Thus, we conclude that LCAR supplementation preserves the key enzymes responsible for maintaining carnitine homeostasis and preserves both cell viability and synaptic transmission after OGD.

## Introduction

Neonatal hypoxic-ischemic encephalopathy (HIE) is a pathological condition that occurs in 1–6 per 1000 live births and results in significant neurodevelopmental disabilities in affected infants [1], [2]. Neonatal HIE occurs when cerebral blood flow is significantly impaired before, during, or immediately after parturition, rendering the brain ischemic. If the ischemia persists, HIE will occur leading to seizures, blindness, severe cognitive deficits, and cerebral palsy characterized by hemiplegia, paraplegia, or quadriplegia [1]. At a molecular level, neonatal ischemia increases glutamate release activating NMDA, AMPA, and kainate receptors elevating cytosolic Ca^2+^. As Ca^2+^ levels increase mitochondrial buffering is overwhelmed, Ca^2+^ is spontaneously released, and the mitochondrial permeability transition pore (MPTP) is activated [3]. MPTP activation depolarizes the mitochondria resulting in a loss of ATP production, an increase in reactive oxygen species (ROS) and damage to cytochromes in the electron transport chain [4]. The increased presence of ROS and Ca^2+^ within the mitochondria also disrupts key enzymes and transporters further exacerbating ATP depletion leading to necrotic and/or apoptotic cell death [2], [3], [4], [5].

A crucial mitochondrial system disrupted by HI is the carnitine pathway [6]. Carnitine facilitates the transport of long chain fatty acids from the cytosol across the mitochondrial matrix for metabolism via beta-oxidation and their utilization in the citric acid cycle [7], [8], [9] Carnitine palmitoyl transferase 1 (CPT1), carnitine acylcarnitine translocase, and carnitine palmitoyl transferase 2 (CPT2) are the 3 key transporters that shuttle fatty acids into the mitochondria as long-chain fatty acylcarnitine esters [7], [9]. Once inside the mitochondrial matrix carnitine removes toxic fatty acyl-CoA metabolites and supplements a pool of free CoA that is actively utilized to maintain acyl-CoA/free CoA homeostasis. During HI, mitochondrial dysfunction causes an accumulation of acyl-CoA moieties that inhibit glycolysis, gluconeogenesis, the citric acid cycle, fatty acid, and protein catabolism [4], [10]. We have previously shown that carnitine homeostasis is disrupted by HI [6] and that the administration of L-carnitine (LCAR) to neonatal rat pups resulted in significant decreases in infarct size 7- and 28-days post HI [6]. However, it is unclear how HI disrupts carnitine homeostasis in the neonatal brain nor is it well understood how the neuroprotection by LCAR supplementation is mediated. To begin to elucidate these mechanisms, we utilized neonatal rat hippocampal slice cultures exposed to oxygen glucose deprivation (OGD) as an in vitro model of neonatal HI to evaluate alterations in carnitine homeostasis, and the effect of LCAR supplementation on mitochondrial function, cell viability, and neuronal signaling.

**Figure 1:**
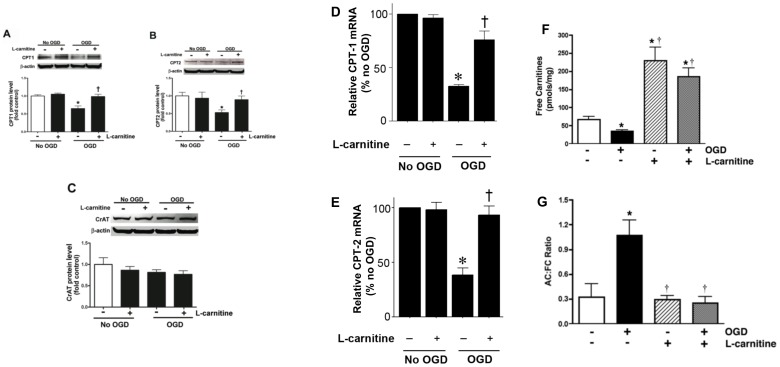
Oxygen glucose deprivation disrupts carnitine homeostasis in rat hippocampal slice cultures. Rat hippocampal slice cultures were exposed to OGD in the presence or absence of L-carnitine (LCAR, 5 mM, 2 h prior to OGD). Slices were harvested 2 h after OGD and Protein extracts (50 µg) were subjected to Western blot analysis to determine effects on CPT1 (A), CPT2 (B), and CrAT (C) protein levels. A representative blot is shown in the inset of each panel. Two hours after OGD total RNA was also isolated and mRNA levels for CTP1 (D) and CTP2 (E) were determined by SYBR Green real-time RT-PCR analyses. Both protein and mRNA expression was normalized using β-actin. In addition, the effect of OGD, in the presence and absence of LCAR, on free carnitine levels (FC, F) and the acylcarnitine (AC): FC ratio (G) was determined. Data are presented as mean ± S.E from 3 independent experiments using 60 pooled slices per experiment. * = P <0.05 vs. no OGD; † = P<0.05 vs. OGD alone.

Our data indicate that OGD decreases CPT1 and CPT2 protein levels and this is prevented by LCAR supplementation. LCAR supplementation also restored the AC∶FC ratio and this attenuated the decrease in mitochondrial dysfunction associated with OGD. Further, the preservation of mitochondrial viability prevented the induction of apoptosis in the slice cultures and preserved the functional activity of synaptic pathways within the hippocampus after OGD.

**Figure 2:**
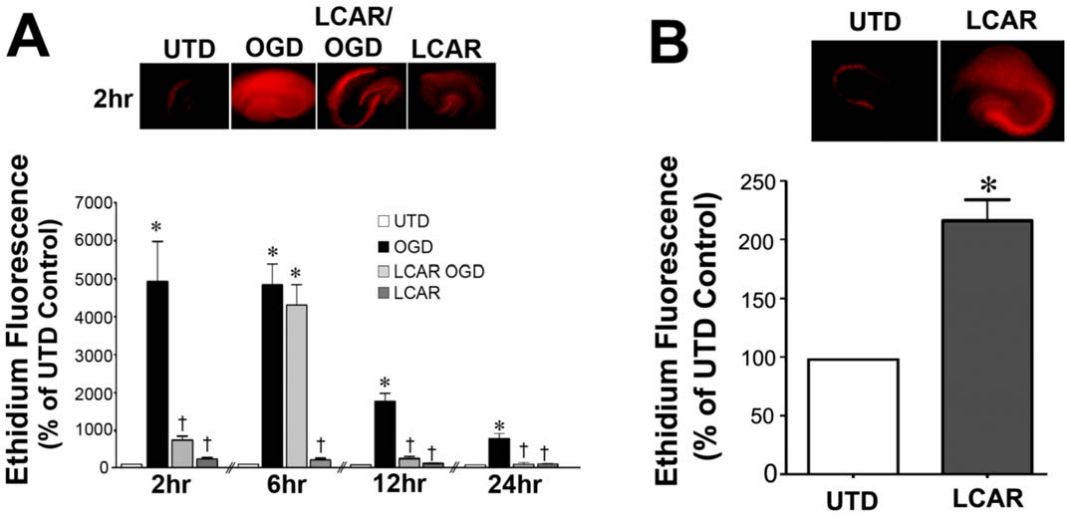
LCAR attenuates the increases in superoxide generation in rat hippocampal slice cultures exposed to oxygen glucose deprivation. Rat hippocampal slice cultures were exposed to OGD in the presence or absence of L-carnitine (LCAR, 5 mM, 2 h prior to OGD). Fluorescent imaging was then utilized to determine changes in DHE oxidation as a measure of total superoxide levels. Slices were then incubated with DHE (20 µM, 20 min) at 2-, 6-, 12- and 24-h after OGD. Representative images are shown for 2 h (A). The effect of LACR pre-treatment alone on cellular superoxide levels was also determined with representative images shown (B). Data are presented as mean ± S.E. using 9–18 slices. * = P<0.05 vs. no OGD; † = P<0.05 vs. OGD alone.

## Materials and Methods

### Ethics Statement

All animal work was conducted according to national guidelines for the ethical treatment of animals in research. All animal protocols were approved by the institutional review boards of the University of Montana and Georgia Health Sciences.

**Figure 3:**
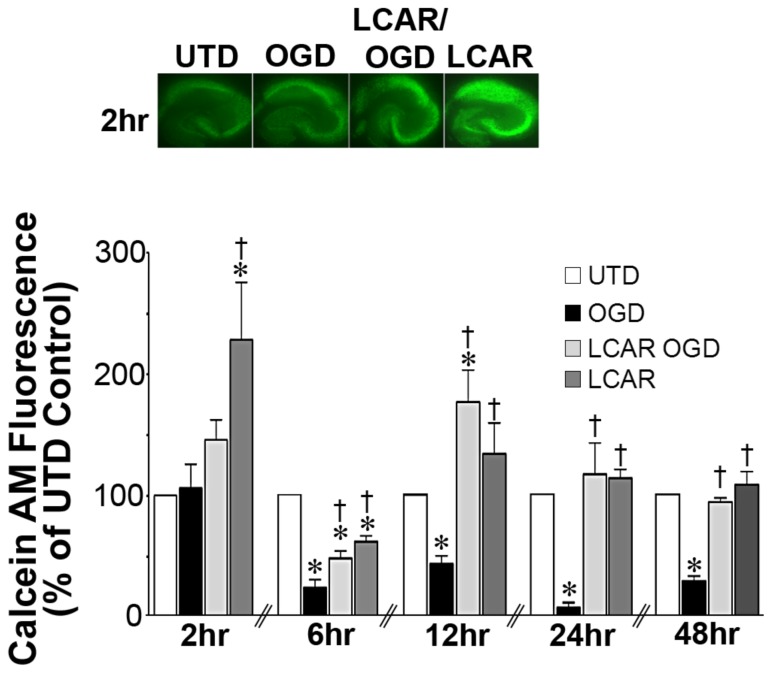
LCAR attenuates the loss of metabolic viability in rat hippocampal slice cultures exposed to oxygen glucose deprivation. Rat hippocampal slice cultures were exposed to OGD in the presence or absence of LCAR (5 mM, 2 h prior to OGD). Slices were then incubated with calcein AM (30 µM, 30 min) to estimate changes in metabolic viability. Calcein AM staining was carried out at 2-, 6-, 12-, 24- and 48-h after OGD. Representative images are shown for 2 h (A). Data are presented as mean ± S.E using 5–9 slices (B). * = P<0.05 vs. no OGD; † = P<0.05 vs. OGD alone.

### Hippocampal Slice Preparation and Induction of Oxygen Glucose Deprivation

Neonatal rats (Sprague-Dawley) at postnatal Day 7 (P7) were decapitated and the hippocampi dissected out under sterile conditions. The hippocampi were cut into 400 μm slices using a McIlwain tissue chopper and individual slices were cultured on Millicell permeable membranes (0.4 μM pore size) in six well plates for 6 days at 37°C in 5% CO_2_. For the first two days, the slices were maintained in a primary plating media (50% DMEM, 25% HBSS, 25% heat inactivated horse serum, 5 mg/mL D-glucose (Sigma), 1 mM Glutamax, 1.5% PenStrep/Fungizone (Invitrogen), and 5 mL of 50X B27 supplement plus anti-oxidants (Invitrogen). On the fourth day in culture, slices were placed in serum-free neurobasal medium (10 mL Neurobasal-A, 200 μL of 50X B27 supplement, 100 μL of 100X Fungizone, and 100 μL of 100X Glutamax). Twenty-four hours prior to experimentation (day 6), the inserts were placed in serum-free neurobasal A media and B27 supplement without antioxidants. Two hours prior to the induction of oxygen-glucose deprivation (OGD), slices were treated with LCAR (5 mM) dissolved in serum-free neurobasal A media and B27 supplement without antioxidants. A glucose free balanced salt solution (BSS) (120 mM NaCl, 5 mM KCl, 1.25 mM NaH_2_PO_4_, 2 mM MgSO_4_, 2 mM CaCl_2_, 25 mM NaHCO_3_, 20 mM HEPES, 25 mM sucrose pH of 7.3) was infused for 1 h with 5% CO_2_/95% nitrogen at approximately 10 L/h. Deoxygenated BSS was placed in a 6 well plate and warmed in a Pro-Ox chamber for 15 minutes with an oxygen feedback sensor that maintained gas levels at 0.1% O_2_. The inserts were then transferred into deoxygenated BSS and placed back into the tank at 0.1% oxygen for 90 min. After OGD, the slices were immediately transferred into pre-warmed serum-free Neurobasal supplemented with B27 without antioxidants in the presence or absence of LCAR (5 mM) under normal O_2_ conditions.

**Figure 4:**
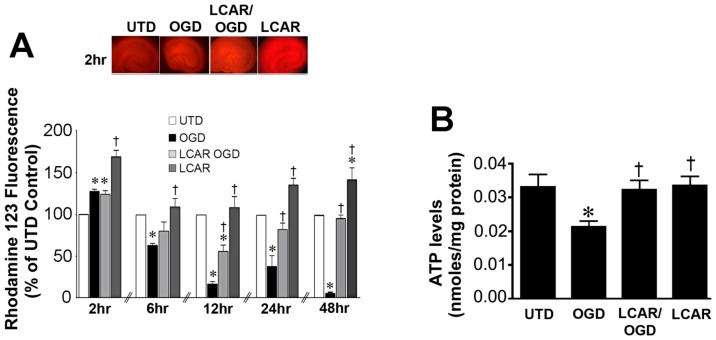
LCAR attenuates the loss of mitochondrial membrane potential in rat hippocampal slice cultures exposed to oxygen glucose deprivation. Rat hippocampal slice cultures were exposed to OGD in the presence or absence of LCAR (5 mM, 2 h prior to OGD). Slices were then incubated with Rhodamine-123 (10 µM, 20 min) 2-, 6-, 12-, 24- and 48-h after OGD to estimate changes in mitochondrial membrane potential. Representative images are shown for 2 h (A). ATP in the slice cultures was also determined (B). Data are presented as mean ± S.E using 7–9 slices. * = P<0.05 vs. no OGD; † = P<0.05 vs. OGD alone.

### Real-time RT-PCR analysis

Real-time RT-PCR was employed to evaluate changes in the levels of CTP-1 and CTP-2 mRNA in response to OGD. All primers used were designed by Primer 3. The sequences were CTP-1: forward 5′- GGAGACAGACACCATCCAACATA-3′; reverse, 5′-AGGTGATGGACTTGTCAAACC-3′; CTP-2: forward, 5′-TCCTCGATCAAGATGGGAAC-3′; reverse, 5′-GATCCTTCATCGGGAAGTCA-3′; β-actin forward, 5′-CTCTTCCAGCCTTCCTTCCT-3′; reverse, 5′-GGGCAGTGATCTCTTTCTGC-3′. Real-time RT-PCR was carried out in two steps. First, total RNA was extracted from cells using the RNeasy kit (Qiagen, Valencia, CA, USA), and 1 μg of total RNA was reverse-transcribed using the QuantiTect Reverse Transcription Kit (Qiagen) in a total volume of 20 μl. Quantitative real-time PCR was conducted on an Mx4000 (Stratagene, La Jolla, CA, USA), using 2 μl of RT product, 12.5 µl of QuantiTect SYBR Green PCR Master Mix (Qiagen), and primers (400 nM) in a total volume of 25 μl. The following thermocycling conditions were employed: 95°C for 10 min, followed by 95°C for 30 s, 55°C for 60 s, and 72°C 30 s for 45 cycles. The threshold cycles (*C*_t_) of a serially diluted control sample were plotted to generate a standard curve. The concentration of each sample was calculated by interpolating its *C*_t_ on the standard curve and then normalized to β-actin (housekeeping gene) mRNA levels.

**Figure 5:**
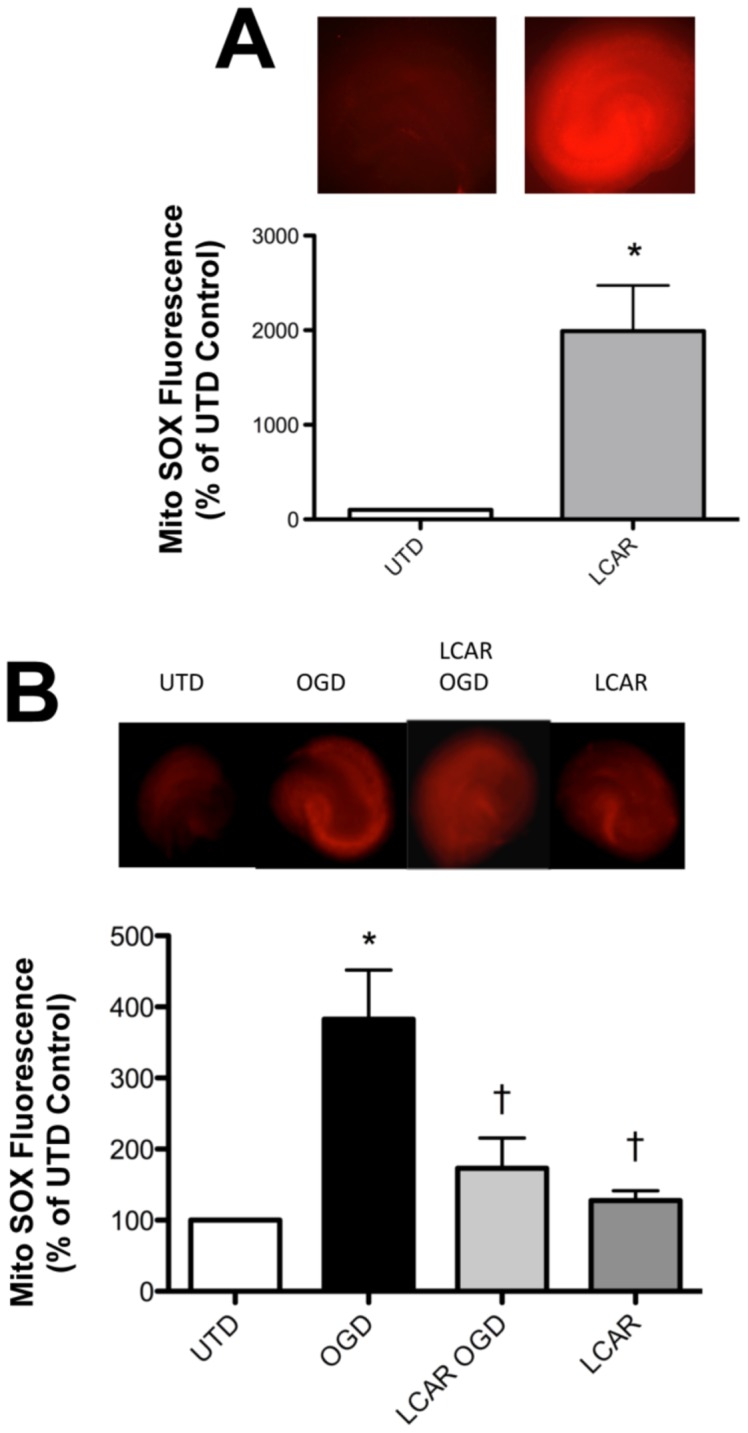
LCAR attenuates mitochondrial superoxide induced by oxygen glucose deprivation in rat hippocampal slice cultures. Rat hippocampal slice cultures were exposed to OGD in the presence or absence of LCAR (5 mM, 2 h prior to OGD). Slices were then incubated with MitoSOX fluorescent dye (40 µM, 30 min) to estimate changes in mitochondrial superoxide levels either after LCAR pre-treatment (A) or 24 h post-OGD (B). Representative images are shown. Data are presented as mean ± S.E using 5–7 slices. * = P<0.05 vs. no OGD; † = P<0.05 vs. OGD alone.

### Western Blot Analysis

Protein extracts were prepared by homogenizing hippocampal slices (4–15 slices were pooled for each determination) in 50 mM Tris-HCl, pH 7.6, 0.5%Triton X-100, 20% glycerol containing Halt^TM^ protease inhibitor cocktail (Pierce Laboratories, Rockford, IL). The extracts were then subjected to centrifugation (15,000 g x 15 min at 4°C). Supernatant fractions were assayed for protein concentration using the Bradford reagent (Bio-Rad, Richmond, CA) and used for Western blot analyses. Protein extracts (50 µg) were separated on Long-Life 4–20% Tris-SDS-Hepes gels (Frenchs Forest, Australia) and electrophoretically transferred to Immuno-Blot PVDF membrane (Bio-Rad Laboratories, Hercules, CA). The membranes were blocked with 5% nonfat dry milk in Tris-buffered saline containing 0.1% Tween 20 (TBST). After blocking, the membranes were probed with antibodies to CrAT (Santa Cruz Biotechnology, Inc.), CPT1 (Affinity Bioreagents), CPT2 (Affinity Bioreagents), HIF-1α (Abcam), AMPKα2 (Millipore), phospho-AMPKαTh172 (Millipore), or cleaved caspase 9 (CC-9, Cell Signaling). Reactive bands were visualized using chemiluminescence (SuperSignal® West Femto Substrate Kit, Pierce Laboratories, Rockford, IL) on a Kodak 440CF image station (Kodak, Rochester, NY). Band intensity was quantified using Kodak 1D image processing software. Each protein was normalized by reprobing with β-actin (Sigma, St.Louis, MO).

**Figure 6:**
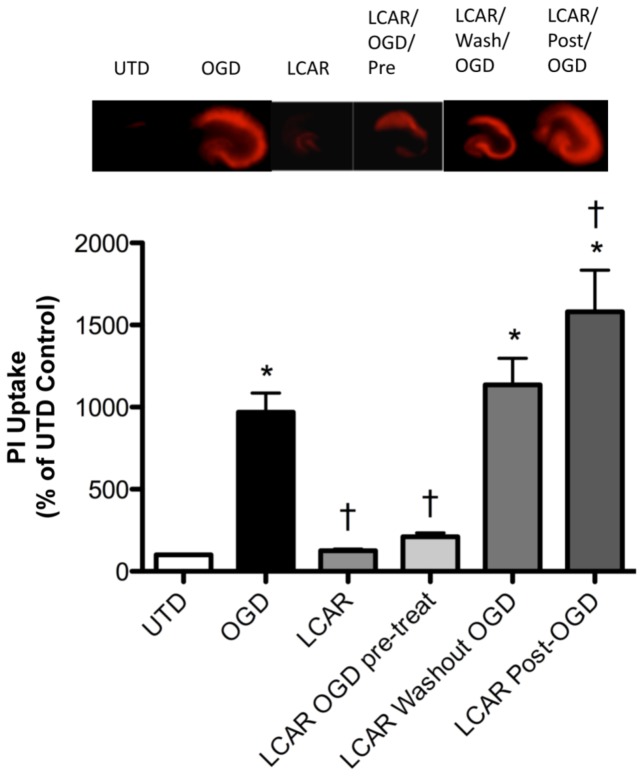
Effect of LCAR supplementation on the neuronal cell death associated with oxygen glucose deprivation in rat hippocampal slice cultures. Rat hippocampal slice cultures were exposed or not to OGD in the presence or absence of LCAR (5 mM) and PI (100 ng/ml). The LCAR was added 2 h prior to OGD and maintained throughout the exposure, washed out after OGD, or added post-OGD. Images were then captured 24 h post-OGD. Representative images are shown. Data are presented as mean ± S.E using 6–8 slices. * = P<0.05 vs. no OGD; † = P<0.05 vs. OGD alone.

### Measurement of Free and Total Carnitines

Detection of carnitines was performed using a Shimadzu UFLC purifier system with a 5 µm Omnispher C18 column (250×4.6 mm OD) and equipped with a RF-10A XL fluorescence detector (Shimadzu, Tokyo, Japan). Total and free carnitines levels were quantified in the hippocampal slices by fluorescence detection at 248 nm (excitation) and 418 nm (emission).

**Figure 7:**
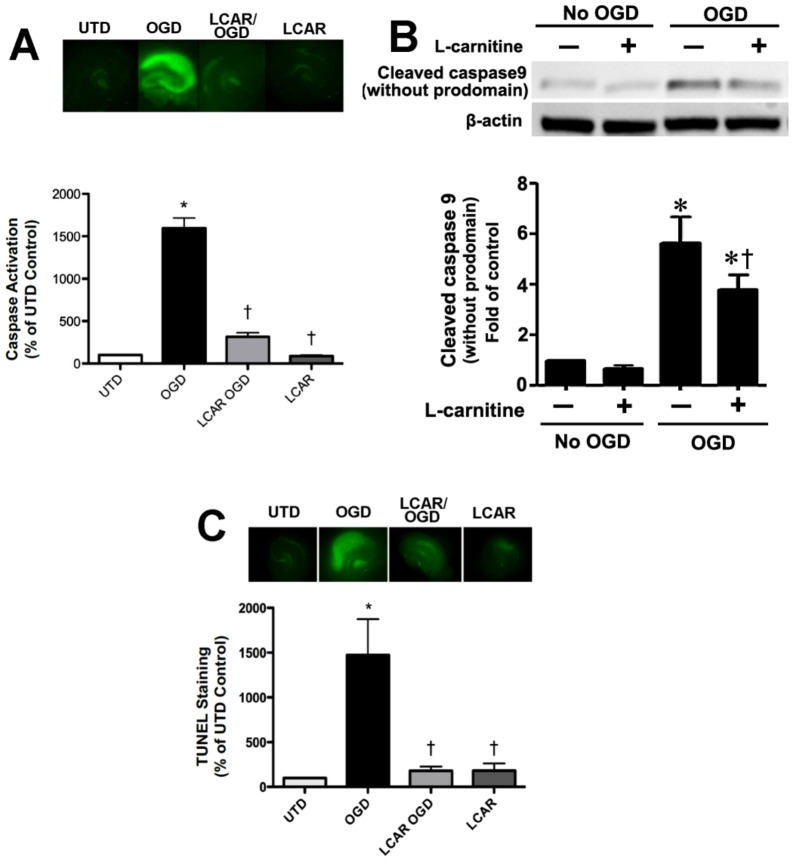
LCAR attenuates the increase in apoptosis induced by oxygen glucose deprivation in rat hippocampal slice cultures. Rat hippocampal slice cultures were exposed to OGD in the presence or absence of LCAR (5 mM, 2 h prior to OGD). After 24 h slices were exposed to the fluorescent caspase activation reagent (10 µM, 20 min) to estimate changes in caspase activity (A). Representative images are shown. Protein extracts (50 µg) were also subjected to Western blot analysis to determine effects on cleaved caspase 9 (CC-9). Each gel was normalized for loading using β-actin. Slices were also analyzed for TUNEL positive nuclei (C). Data are presented as mean ≤ S.E using 8 slices. * = P<0.05 vs. no OGD; † = P<0.05 vs. OGD alone.

#### Homogenate preparation

Hippocampal slices (60 per group) were weighed and homogenized in 1∶4 volumes of 0.5 M HClO_4_. All samples were then centrifuged at 12,000 rpm (∼13,000 g) in an microcentrifue for 20 min. The pellet was discarded and the supernatant neutralized to pH 7.0 with 3 M KHCO_3_. These were subsequently used as homogenates.

**Figure 8:**
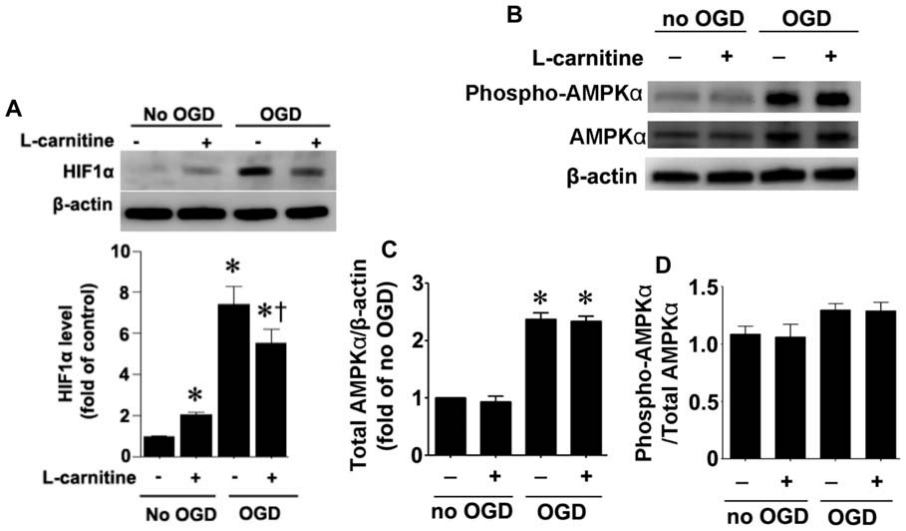
Affect of oxygen glucose deprivation and LCAR on survival factors in rat hippocampal slice cultures exposed to oxygen glucose deprivation. Rat hippocampal slice cultures were exposed to OGD in the presence or absence of L-carnitine (LCAR, 5 mM, 2 h prior to OGD). Slices were harvested 2 h after OGD and Protein extracts (50µg) were subjected to Western blot analysis to determine effects on HIF-1α (A), AMPKα2 (B & C), and phospho-AMPK-Th172 (B & D) protein levels. Total AMPKα2 protein was normalized using β-actin. Data are presented as mean ± S.E from 3 independent experiments using 60 pooled slices per experiment. * = P<0.05 vs. no OGD; † = P<0.05 vs. OGD alone.

#### Sample purification and derivatization before HPLC detection

For free carnitine (L-carnitine and acetyl L-carnitine) determination- 300 µl homogenate, 100 µl water and 100 µl of internal standard (Sigma ST 1093) were mixed. For total carnitine determination- 300 µl homogenate were hydrolyzed with 0.3 M KOH, heated at 80°C for 20 min, pH neutralized using perchloric acid and 100 µl internal standard was added. All samples were purified using solid phase extraction columns, SAX 100 mg/ml (Varian, Harbor City, CA) and derivatized using 1-aminoanthracene in presence of EDCI (catalyst) and kept at 30°C for 1 hour to complete reaction of carnitines. Separation on HPLC was carried out with an isocratic elution in 0.1 M Tris-acetate buffer (pH 3.5): acetonitrile (68∶32, v/v) at a flow rate of 0.9 ml/min.

**Figure 9:**
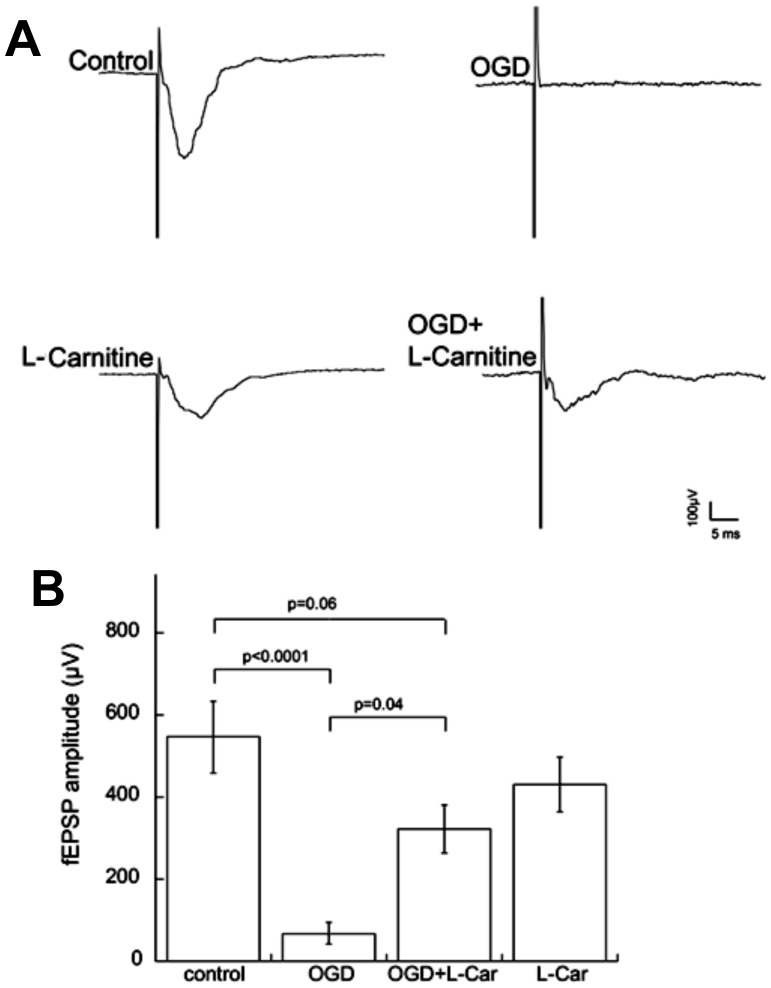
LCAR maintains synaptic viability in the CA1 region after oxygen glucose deprivation. Rat hippocampal slice cultures were exposed to OGD in the presence or absence of LCAR (5 mM, 2 h prior to OGD). Representative recordings of Schaffer collateral-CA1 pyramidal neuron field EPSPs made 48 h following OGD (A). Data are presented as mean ± S.E from 4 independent experiments using 3–5 slices per experiment (B). * = P<0.05 vs. no OGD; † = P<0.05 vs. OGD alone.

### Measurement of global and Mitochondrial Superoxide levels

Total superoxide levels were estimated by dihydroethidium (DHE) oxidation as we have previously described [11], [12]. Mitochondrial superoxide levels were estimated using the oxidation of the MitoSOX fluorescent dye as we have described [13]. Briefly, rat hippocampal slices were incubated with DHE (20 μM, Molecular Probes) or MITOSOX (40 μM, Molecular Probes) for 20–30 min, washed 3 times with pre-warmed PBS without calcium or magnesium, and captured on an Olympus IX51 fluorescent microscope at 518 nm (excitation) and 518 nm (emission) and the 4X objective lens. The fluorescent intensity was quantified in Image Pro Plus using the IOD (average density X area) measurement. All values obtained were normalized to the untreated control mean and expressed as a percent change.

### Quantification of Slice Culture Cell Death using Propidium Iodide uptake

Propidium iodide (PI, Molecular Probes) uptake was utilized to determine decreases in overall neuronal death associated with OGD. Neonatal hippocampal brain slices were incubated with PI (100 ng/ml) for 24 h then washed 3 times with pre-warmed PBS without calcium or magnesium. Slice cultures were then examined prior to OGD using fluorescent microscopy with detection at 518 nm (excitation) and 604nm (emission). Slices showing distinct PI labeling at this stage were deemed damaged and were then excluded from further study. Slice culture images were obtained at 0-, 4- 8-, and 24 h after OGD using a 10-bit monochrome fluorescence camera (HAMAMATSU Digital Camera C4742-95, Hamamatsu, Japan), and processed with Image-Pro Plus6.0 (Media Cybernetics, Maryland, U.S.A.). The exposure time was set at 200 ms, using a 4x magnification to capture the entire slice. The evaluation of cell death was performed using a modification of the method of Cronberg [14]. The fluorescent intensity was reported using IOD (average density X area) measurement. All values obtained were normalized to the untreated control mean and expressed as percent change.

### Estimation of Mitochondrial Metabolic Viability

The functional activity of mitochondrial esterases was determined by Calcein AM staining. Rat hippocampal slices were incubated with Calcein AM (30 μM, Molecular Probes) for 30 min, washed 3 times with pre-warmed PBS without calcium or magnesium, and fluorescent images captured using 506 nm (excitation) and 529 nm (emission) and the 4X objective lens. The fluorescent intensity of captured images was quantified in Image Pro Plus using the IOD (average density X area) function. All values obtained were normalized to the untreated control mean and expressed as a percent change.

### Measurement of Mitochondrial Membrane Potential

Mitochondrial membrane potential (MMP) was measured over time by staining rat hippocampal slice cultures with Rhodamine-123 (Molecular Probes). Slices were preloaded with Rhodamine-123 (10 μM) 20 min prior to image capture. Slices were washed 3 times with pre-warmed PBS without calcium or magnesium and images captured using fluorescent microscopy with detection at 518 nm (excitation) and 604 nm (emission). The fluorescent intensity of images was quantified in Image Pro Plus using the IOD (average density X area) function. All values obtained were normalized to the untreated control mean and expressed as a percent change.

### Measurement of Caspase Activation

Twenty-four hours post-OGD caspase activation was measured by exposing slices to the caspase activation reagent (10 µM, Promega) for 20 min. Slices were then washed 3 times with pre-warmed PBS without calcium or magnesium, and images captured using fluorescent microscopy with detection at 506 nm (excitation) and 529 nm (emission) using the 4X objective lens. The fluorescent intensity was quantified in Image Pro Plus using IOD (average density x area) function. All values obtained were normalized to the untreated control mean and expressed as a percent change.

### Measurement of Apoptotic Cell Death

Apoptotic neuronal death was measured by TUNEL staining (Promega). Slices were fixed in 4% paraformaldehyde for 20 min at room temperature, rinsed in PBS three times and removed from the Millicell inserts, placed on glass slides. and processed according to the manufacturer's instructions. Images were captured using fluorescent microscopy with detection at 506 nm (excitation) and 529 nm (emission) using the 4X objective lens and analyzed using ImagePro software. All values obtained were normalized to the untreated control mean and expressed as a percent change.

### Electrophysiology/field recording

Slices were separated and placed in artificial cerebral spinal fluid (ACSF) containing (in mM): 126 NaCl, 2.5 KCl, 1.2 MgCl_2_, 2.4 CaCl_2_, 1.2 NaH_2_PO_4_, 11.4 glucose, and 21.4 NaHCO_3_, saturated with 95% O_2_ and 5% CO_2_ (pH 7.3), and maintained at 30°C. For recording, slices were transferred to a submersion type recording chamber constantly perfused (1.9 ml/min) with saturated ACSF at 30°C. Field excitatory post synaptic potentials (fEPSPs) were recorded in stratum radiatum of CA1 using glass stimulating and recording microelectrodes filled with HEPES buffered ACSF (pH 7.3). EPSPs were monitored at 0.05 Hz and were recorded using a Molecular Devices amplifiers interfaced to a Digidata 1200 analog/digital converter. Control and analysis was performed using AxographX (Version 1.0) or pClamp9 (Molecular Devices) software.

### Statistical Analysis

All data was analyzed utilizing Prism software (GraphPad Software, Inc., La Jolla, CA). One-way ANOVA with Tukey's post-hoc was used to determine statistical significance between groups. Two-tailed, unpaired t-test was used to determine specific differences between single groups. A p value of <0.05 was considered significant, and slices that were greater than ± 4 standard deviations from the mean were considered outliers and omitted from statistical analyses.

## Results

### L-carnitine supplementation ameliorates the OGD-induced disruption of carnitine homeostasis

CPT1 and CPT2 facilitate the transport of long-chain fatty acids from cytosol into the mitochondrial matrix, the site of β-oxidation. Carnitine acetyl transferase (CrAT), which resides in the matrix, is able to reconvert the short- and medium chain acyl-CoAs into acylcarnitines using intra-mitochondrial carnitine. Using hippocampal slice cultures exposed to oxygen glucose deprivation (OGD), we initially examined the effect of OGD on the protein levels of these three important mitochondrial proteins involved in carnitine metabolism. Our data indicate that OGD significantly decreases both CPT1 (∼40% decrease, Fig. 1 A) and CPT2 (∼50% decrease, Fig. 1 B) protein levels and this decrease was ameliorated by LCAR treatment (Fig. 1 A & B). We found no effect of OGD or LCAR on the levels of the CrAT protein (Fig. 1 C). Changes in CPT1 and CPT2 protein were also reflected at the mRNA level (D & E). To determine if the OGD mediated alterations in CPT1 and CPT2 resulted in disruption of cellular carnitine homeostasis, we measured free carnitine (FC) levels and determined the acylcarnitine: free carnitine (AC∶FC) ratio using HPLC analysis. Our data indicate that OGD alone caused a significant decrease in FC levels (Fig. 1 F). LCAR supplementation prior to OGD increased the levels of FC (Fig. 1 F). Further, we found that OGD significantly increased the AC∶FC ratio. However, this effect was prevented by LCAR (Fig. 1 G). The preservation of carnitine metabolism correlated with a decrease in total cellular oxidative stress post-OGD (as determined by DHE oxidation) (Fig. 2 A). However, DHE oxidation was significantly increased by LCAR treatment prior to OGD (Fig. 2 B).

### LCAR supplementation maintains mitochondrial function in rat hippocampal slice cultures exposed to OGD

To measure the effect of LCAR supplementation on mitochondrial function after OGD, cultures were assayed with Calcein AM, a fluorogenic esterase substrate that is retained and cleaved in neurons that are metabolically active. Utilizing this dye, the effect of mitochondrial metabolic viability was assayed in slice cultures at 2–48 h (Fig. 3) after OGD. Slices exposed to OGD exhibited an irreversible decrease in metabolic activity (decreased fluorescence) that was attenuated in the LCAR treated slices (Fig. 3). LCAR treatment in non-OGD slices also resulted in an increase in Calcein AM fluorescence suggesting LCAR not only prevents mitochondrial dysfunction but also increases metabolic activity prior to OGD (Fig. 3 A). To further evaluate the effect of LCAR supplementation on mitochondrial function we next examined the effect on mitochondrial membrane potential (MMP) after OGD. Utilizing Rhodamine-123 fluorescent staining, mitochondrial membrane potential was measured at 2–48 h (Fig. 4 A) post-OGD. At 2 h Rhodamine-123 fluorescence was increased in all groups compared to control slices (Fig. 4 A). However, Rhodamine-123 staining was significantly decreased at 6-, 12-, 24- and 48-h post-OGD indicating mitochondrial depolarization in neurons (Fig. 4 A). In contrast, carnitine supplemented slices exposed to OGD showed MMP depolarization 12-h post-OGD (Fig. 4 A), but demonstrated a complete recovery (basal repolarization compared to non-OGD control slices) of MMP at 24- and 48-h post-OGD (Fig. 4 A). Slice cultures exposed to LCAR alone had a significant increase in Rhodamine-123 staining at all time points tested. This finding suggests LCAR treatment significantly increased MMP above basal levels and this effect correlated with a preservation of ATP levels in the slice culture (Fig. 4 B). Further, we found that although LCAR supplementation increased mitochondrial-derived ROS prior to OGD (as determined using MitoSOX fluorescence) (Fig. 5 A) there was a significant decrease in mitochondrial superoxide generation 24 h post-OGD (Fig. 5 B).

### LCAR supplementation reduces neuronal cell death in rat hippocampal slice cultures exposed to OGD

We next determined if the LCAR-mediated preservation of mitochondrial function could attenuate the neuronal cell death associated with OGD. Our data indicate that LCAR supplementation prior to OGD decreased PI uptake in slice cultures exposed to OGD (Fig. 6). However, this protection was lost if the LCAR was removed prior to OGD (Fig. 6). Further, if LCAR was added only post-OGD the PI uptake was enhanced indicating increased cell death was occurring (Fig. 6). We next evaluated the effect of LCAR supplementation on apoptotic signaling events. Using a fluorescent assay for pan-caspase activation, we found that LCAR supplementation significantly inhibited overall caspase activation 24 h post-reperfusion (Fig. 7 A). To further elucidate this effect, we examined mitochondrially cleaved (activated) caspase 9 (CC-9). CC-9 is a major component of mitochondrial mediated caspase activation after hypoxic insult [5]. Using Western blot analysis, we found that LCAR supplementation significantly decreased the levels of CC-9 (Fig. 7 B). To ensure that LCAR mediated decreases in caspase activation correlated with decreased apoptosis, fixed hippocampal slices 24 h post-OGD were assayed for TUNEL staining. Our data indicate that LCAR supplementation significantly decreased TUNEL staining of neurons 24 h post reperfusion (Fig. 7 C).

### LCAR supplementation attenuates HIF-1α induction in rat hippocampal slice cultures exposed to OGD

To determine if LCAR was modulating the levels of other proteins that could modulate neuronal survival we evaluated changes in HIF-1α and AMPKα2. Our data indicate that OGD significantly increased both HIF-1α (Fig. 8 A) and AMPKα2 (Fig. 8 B & C). LCAR supplementation significantly decreased the OGD-mediated increase in HIF-1α (Fig. 8 A) but had no affect on the increase in AMPKα2 (Fig. 8 B & C). LACR supplementation in the absence of OGD also increased HIF-1α but to a much lesser extent than OGD (Fig. 8 A). In addition, although phospho-AMPK-Th172 levels were increased by OGD (Fig. 8 B) when normalized to total AMPKα2 (Fig. 8 B & C) there was no relative change (Fig. 8 B & D). Again LCAR supplementation had no affect of the relative levels of phospho-AMPK-Th172 (Fig. 8 D).

### LCAR supplementation protects synaptic transmission in rat hippocampal slice cultures exposed to OGD

While experimental data obtained from slice cultures demonstrated a link between LCAR treatment and decreased cell death, these data did not assess the viability and synaptic function of LCAR treated neurons. To address this issue, LCAR supplemented cultures were exposed to OGD and field recording experiments were performed to measure excitatory transmission. Measurement of synaptic transmission in slice cultures showed that OGD significantly decreased field excitatory postsynaptic potential (fEPSP) amplitudes (Fig. 9 A). In contrast, LCAR treated slices protected synaptic viability, as responses following OGD in the presence of LCAR were not significantly different from the untreated controls 48 h post reperfusion (Fig. 9 B). These data suggest LCAR not only decreased neuronal death, but also preserved synaptic transmission after OGD.

## Discussion

The mitochondrial membrane is impermeable to long-chain fatty acids and the transport of these fatty acids across mitochondrial membrane is mediated by the enzymes: CPT1 and CPT2 [4], [15], [16]. CPT1 is localized in the outer mitochondrial membrane and conjugates carnitine with long-chain acyl-CoA to make acylcarnitines. At the inner mitochondrial membrane, CPT2 transesterifies acylcarnitines back to free carnitine and long-chain acyl CoA [6], [17], [18]. Thus, transfer of fatty acids into the mitochondria for β-oxidation is dependent on the function of CPT1 and CPT2 and therefore the decreases in their expression mediated by OGD would disrupt β-oxidation. This could explain the disruption in energy metabolism in the mitochondria as fatty acids are the most important metabolic fuel under fasting or stress conditions, especially in neonates. Further, prior studies have found that low FC levels and high AC levels in neonates exposed to HI [19], [20]. Although a recent study has shown that mitochondrial CPT1 level and β-oxidation of fatty acids were significantly reduced in muscle and liver of rats exposed to cold-hypobaric hypoxic environment [21] this is the first study indicating that OGD decreases the expression of CPT1 and CPT 2 in organotypic slice cultures. However, the regulation of CPT1 by oxygen may be cell type dependent as a recent study has shown that CPT1 is increased by hypoxia or glucose deprivation in tumor cells [22]. The increase in CPT1 involved AMPKα. However, in our organotypic slice cultures although we observed an increase in AMPKα protein levels there were no changes in phopsho-AMPK-Th172 and a decrease in CPT1 again suggesting cell specific events may be involved.

HI in the newborn disrupts energy metabolism in mitochondria and it is likely that LCAR limits the reperfusion injury after hypoxia by preventing long-chain acyl-CoA accumulation and subsequent production of ROS by damaged mitochondria. In previous studies it has been shown that LCAR treatment increases cellular respiration, and, as a byproduct, increased superoxide production [23], [24]. Our data confirm this observation as we found DHE oxidation to be significantly elevated in LCAR treated slices prior to exposure to OGD. LCAR supplementation prior to OGD did not increase basal ATP levels. However, during post-reperfusion LCAR supplementation significantly reduced DHE oxidation and maintained basal levels of ATP, suggesting that there was a reduction in oxidative stress and a preservation of metabolic viability. Furthermore, using MitoSOX to estimate mitochondrial levels of superoxide we found the same result: carnitine treatment increased mitochondrial superoxide levels prior to OGD while reducing mitochondrial superoxide, enhancing mitochondrial function and attenuating neuronal death post-OGD.

Pre-treatment with LCAR increased markers of mitochondrial metabolic viability and mitochondrial membrane potential (MMP) prior to OGD. This is in agreement with previous studies from Ames et al demonstrating LCAR treatment increases ATP and ROS within the mitochondria as a result of increased fatty acid transport [24], [25], [26], [27]. It may appear paradoxical that increasing ROS prior to OGD would have a neuroprotective effect as it is widely accepted that neuronal mitochondria lack robust anti-oxidant defense systems and are highly sensitive to lipid membrane damage associated with increased ROS levels [28]. However, it is possible that LCAR pre-treatment, by combining increases in ATP and ROS under basal conditions, may be acting as a type of ischemic pre-conditioning in which neurons exposed to a mild insult are protected against a full insult. Support for LCAR-mediated pre-conditioning also comes from our data in which we found that the addition of LCAR post-OGD exacerbated neuronal cell death. If LCAR causes an oxidative insult, albeit a mild form, this would be deleterious in the presence of a severe insult. Although our data do not allow us to delineate the mechanism by which this pre-conditioning is occurring, it may be mediated by an LCAR-dependent increase in anti-oxidant enzyme capacity. Studies have demonstrated that mild ischemia can upregulate SOD1, SOD2 and catalase in hippocampal tissue [29], [30]. Blocking this effect with an antisense to SOD1 significantly decreases the neuroprotective effect [31], [32], [33]. Thus, LCAR may exert its protective effect by increasing antioxidant expression in the mitochondria.

Our data also demonstrated that the increase in HIF-1α induced by OGD was attenuated by LCAR supplementation. HIF-1α was increased in presence of LCAR, but was significantly lower when compared to slices exposed to OGD. The role of HIF-1α in cerebral ischemic events is complex. As a hypoxia induced transcription factor, HIF-1 allows the cellular adaptation to low oxygen conditions via the regulation of multiple target genes, some of which are involved in glucose metabolism and transport. HIF-1α can induce a glycolytic shift (the Warburg effect) that allows cells to generate ATP independently of oxidative phosphorylation in the mitochondria [34]. This may explain our finding that ATP levels are maintained after OGD in LCAR supplemented slice cultures after OGD. In contrast, studies have shown that HIF-1 plays a detrimental role in ischemic reperfusion injury. HIF-1 has been shown to induce the pro-apoptotic factors which can lead to mitochondrial dysfunction and cell death [35]. The duality of HIF-1 may be related both to the level of HIF-1 protein in a cell and the duration of the induction [35]. Thus, the modest increase in HIF-1 induced by LCAR prior to OGD may activate protective signaling pathways that, by attenuating the OGD-mediated large increase in HIF-1, may preserve neuronal function. However, further, studies involving the modulation of HIF-1 activity will be required to test this hypothesis.

Our data also indicated that LCAR exerted a biphasic effect on MMP. MMP levels were found to decrease 6- and 12 h post-reperfusion, but returned to basal levels at 24- and 48-h. This finding suggests LCAR treated cultures are capable of selectively modulating MMP after OGD. This finding could be important as synaptic transmission is rapidly attenuated by HI and after reperfusion it briefly returns only to decrease as the secondary phase of energy depletion occurs [36], [37]. Previously it has been demonstrated that a percentage of neurons exposed to mild HI will not undergo cell death, but are unable to generate functional EPSP's due to acute mitochondrial dysfunction and ATP depletion [38], [39]. However, our data demonstrate that LCAR supplemented slices generated considerably more robust EPSP amplitudes (∼300 μV vs. 75 µV) after OGD and these did not significantly differ from control slices. The preservation of the synaptic transmission in LCAR exposed slices after OGD may be linked to its ability to restore mitochondrial function 24 h post-reperfusion. Our data also show that LCAR attenuates the increase in cleaved caspase 9 (CC-9) levels after OGD. Thus, it is unlikely that activation of the mitochondrial permeability transition pore (MPTP) is occurring in LCAR treated slices [15], [40]. Indeed when the mitochondrial membrane is disrupted the release of cytochrome C leads to increased levels of CC-9 [40]. A possible explanation for the decrease and return of MMP may be linked to activation of mitochondrial uncoupling proteins (UCP's). UCP's are a class of reversible protein pores that serve to uncouple (reduce) the proton motive force by allowing protons to pass back into the inner mitochondrial membrane without generating ATP and increasing ROS levels [41], [42]. This activity has been shown to increase the efflux of calcium, and exert a neuroprotective effect in neurons exposed to OGD [41]. Activation of UCP's has been shown to significantly decrease ROS levels, reduce membrane damage, and induce mitochondrial fission as a compensatory effect [41], [43]. However, future studies will be required to determine if UCP proteins are involved in the neuroprotective effect exerted by LCAR.

In conclusion our data indicate that LCAR supplementation acts to prevent the disruption of carnitine homeostasis through two interconnected pathways. It prevents the OGD-mediated decrease in CPT1 and CPT2 expression and induces an ischemic preconditioning effect in neurons that protects the MMP and prevents the induction in apoptosis during the reperfusion injury post-OGD. As neonatal HI is marked by profound mitochondrial dysfunction that results in neuronal loss and neurological deficits, we speculate that as LCAR represents a compound that prevents mitochondrial dysfunction. Thus, it may have significant clinical relevance in treating neonatal HI. It can be administered both to pregnant females and infants in high doses with no measureable side effects or toxicity [44], [45]. As a possible prophylaxis for neonatal HI, we suggest that carnitine is a neuroprotective compound that warrants further investigation.

## References

[1] Ambalavanan N, Carlo WA, Shankaran S, Bann CM, Emrich SL, et al (2006) Predicting outcomes of neonates diagnosed with hypoxemic-ischemic encephalopathy. Pediatrics 118: 2084–2093. 17079582 10.1542/peds.2006-1591

[2] McLean C, Ferriero D (2004) Mechanisms of hypoxic-ischemic injury in the term infant. Semin Perinatol 28: 425–432. 15693399 10.1053/j.semperi.2004.10.005

[3] Budd SL (1998) Mechanisms of neuronal damage in brain hypoxia/ischemia: focus on the role of mitochondrial calcium accumulation. Pharmacol Ther 80: 203–229. 9839772 10.1016/s0163-7258(98)00029-1

[4] Vallee L, Fontaine M, Nuyts JP, Ricart G, Krivosic I, et al (1994) Stroke, hemiparesis and deficient mitochondrial beta-oxidation. Eur J Pediatr 153: 598–603. 7957409 10.1007/BF02190669

[5] Northington FJ, Graham EM, Martin LJ (2005) Apoptosis in perinatal hypoxic-ischemic brain injury: how important is it and should it be inhibited? Brain Res Brain Res Rev 50: 244–257. 16216332 10.1016/j.brainresrev.2005.07.003

[6] Wainwright MS, Mannix MK, Brown J, Stumpf DA (2003) L-carnitine reduces brain injury after hypoxia-ischemia in newborn rats. Pediatr Res 54: 688–695. 12904603 10.1203/01.PDR.0000085036.07561.9C

[7] Hagen TM, Ingersoll RT, Wehr CM, Lykkesfeldt J, Vinarsky V, et al (1998) Acetyl-L-carnitine fed to old rats partially restores mitochondrial function and ambulatory activity. Proc Natl Acad Sci U S A 95: 9562–9566. 9689120 10.1073/pnas.95.16.9562 PMC21378

[8] Liu J, Atamna H, Kuratsune H, Ames BN (2002) Delaying brain mitochondrial decay and aging with mitochondrial antioxidants and metabolites. Ann N Y Acad Sci 959: 133–166. 11976193 10.1111/j.1749-6632.2002.tb02090.x

[9] Ramsay RR, Zammit VA (2004) Carnitine acyltransferases and their influence on CoA pools in health and disease. Mol Aspects Med 25: 475–493. 15363637 10.1016/j.mam.2004.06.002

[10] Nadtochiy SM, Tompkins AJ, Brookes PS (2006) Different mechanisms of mitochondrial proton leak in ischaemia/reperfusion injury and preconditioning: implications for pathology and cardioprotection. Biochem J 395: 611–618. 16436046 10.1042/BJ20051927 PMC1462692

[11] Grobe AC, Wells SM, Benavidez E, Oishi P, Azakie A, et al (2006) Increased oxidative stress in lambs with increased pulmonary blood flow and pulmonary hypertension: role of NADPH oxidase and endothelial NO synthase. Am J Physiol Lung Cell Mol Physiol 290: L1069–1077. 16684951 10.1152/ajplung.00408.2005

[12] Brennan L, Steinhorn RH, Wedgwood S, Mata-Greenwood E, Roark EA, et al (2003) Increased superoxide generation is associated with pulmonary hypertension in fetal lambs: A role for NADPH oxidase. Circ Res 92: 683–691. 12609968 10.1161/01.RES.0000063424.28903.BB

[13] Sud N, Wells SM, Sharma S, Wiseman DA, Wilham J, et al (2008) Asymmetric dimethylarginine inhibits HSP90 activity in pulmonary arterial endothelial cells: role of mitochondrial dysfunction. Am J Physiol Cell Physiol 294: C1407–1418. 18385287 10.1152/ajpcell.00384.2007 PMC3815615

[14] Cronberg T, Rytter A, Asztely F, Soder A, Wieloch T (2004) Glucose but Not Lactate in Combination With Acidosis Aggravates Ischemic Neuronal Death In Vitro. Stroke 35: 753–757. 14963271 10.1161/01.STR.0000117576.09512.32

[15] Al-Majed AA, Sayed-Ahmed MM, Al-Omar FA, Al-Yahya AA, Aleisa AM, et al (2006) Carnitine esters prevent oxidative stress damage and energy depletion following transient forebrain ischaemia in the rat hippocampus. Clin Exp Pharmacol Physiol 33: 725–733. 16895547 10.1111/j.1440-1681.2006.04425.x

[16] Ames BN (2003) The metabolic tune-up: metabolic harmony and disease prevention. J Nutr 133: 1544S–1548S. 12730462 10.1093/jn/133.5.1544S

[17] Pogatsa G (2001) Metabolic energy metabolism in diabetes: therapeutic implications. Coron Artery Dis 12 Suppl 1S29–33. 11286305

[18] Hwang YC, Bakr S, Ramasamy R, Bergmann SR (2002) Relative importance of enhanced glucose uptake versus attenuation of long-chain acyl carnitines in protecting ischemic myocardium. Coron Artery Dis 13: 313–318. 12436025 10.1097/00019501-200209000-00002

[19] Cam H, Yildirim B, Aydin A, Say A (2005) Carnitine levels in neonatal hypoxia. J Trop Pediatr 51: 106–108. 15677368 10.1093/tropej/fmh089

[20] Bayes R, Campoy C, Goicoechea A, Peinado JM, Pedrosa T, et al (2001) Role of intrapartum hypoxia in carnitine nutritional status during the early neonatal period. Early Hum Dev 65 Suppl: S103–110 10.1016/s0378-3782(01)00212-2 11755041

[21] Dutta A, Vats P, Singh VK, Sharma YK, Singh SN, et al (2009) Impairment of mitochondrial beta-oxidation in rats under cold-hypoxic environment. Int J Biometeorol 53: 397–407. 19396472 10.1007/s00484-009-0224-5

[22] Zaugg K, Yao Y, Reilly PT, Kannan K, Kiarash R, et al (2011) Carnitine palmitoyltransferase 1C promotes cell survival and tumor growth under conditions of metabolic stress. Genes Dev 25: 1041–1051. 21576264 10.1101/gad.1987211 PMC3093120

[23] Shigenaga MK, Hagen TM, Ames BN (1994) Oxidative damage and mitochondrial decay in aging. Proc Natl Acad Sci U S A 91: 10771–10778. 7971961 10.1073/pnas.91.23.10771 PMC45108

[24] Long J, Gao F, Tong L, Cotman CW, Ames BN, et al (2009) Mitochondrial decay in the brains of old rats: ameliorating effect of alpha-lipoic acid and acetyl-L-carnitine. Neurochem Res 34: 755–763. 18846423 10.1007/s11064-008-9850-2 PMC2790461

[25] Hagen TM, Liu J, Lykkesfeldt J, Wehr CM, Ingersoll RT, et al (2002) Feeding acetyl-L-carnitine and lipoic acid to old rats significantly improves metabolic function while decreasing oxidative stress. Proc Natl Acad Sci U S A 99: 1870–1875. 11854487 10.1073/pnas.261708898 PMC122286

[26] Ames BN (1998) Micronutrients prevent cancer and delay aging. Toxicol Lett 102–103: 5–18. 10.1016/s0378-4274(98)00269-0 10022226

[27] Liu J, Head E, Gharib AM, Yuan W, Ingersoll RT, et al (2002) Memory loss in old rats is associated with brain mitochondrial decay and RNA/DNA oxidation: partial reversal by feeding acetyl-L-carnitine and/or R-alpha -lipoic acid. Proc Natl Acad Sci U S A 99: 2356–2361. 11854529 10.1073/pnas.261709299 PMC122369

[28] Berger RP, Adelson PD, Richichi R, Kochanek PM (2006) Serum biomarkers after traumatic and hypoxemic brain injuries: insight into the biochemical response of the pediatric brain to inflicted brain injury. Dev Neurosci 28: 327–335. 16943655 10.1159/000094158

[29] Burda J, Danielisova V, Nemethova M, Gottlieb M, Kravcukova P, et al (2009) Postconditioning and anticonditioning: possibilities to interfere to evoked apoptosis. Cell Mol Neurobiol 29: 821–825. 19259808 10.1007/s10571-009-9363-9 PMC11505781

[30] Domorakova I, Mechirova E, Dankova M, Danielisova V, Burda J (2009) Effect of antioxidant treatment in global ischemia and ischemic postconditioning in the rat hippocampus. Cell Mol Neurobiol 29: 837–844. 19259806 10.1007/s10571-009-9365-7 PMC11506344

[31] Hoshida S, Yamashita N, Otsu K, Hori M (2002) The importance of manganese superoxide dismutase in delayed preconditioning: involvement of reactive oxygen species and cytokines. Cardiovasc Res 55: 495–505. 12160946 10.1016/s0008-6363(02)00337-1

[32] Hoshida S, Kuzuya T, Fuji H, Yamashita N, Oe H, et al (1993) Sublethal ischemia alters myocardial antioxidant activity in canine heart. Am J Physiol 264: H33–39. 8430858 10.1152/ajpheart.1993.264.1.H33

[33] Hoshida S, Kuzuya T, Yamashita N, Oe H, Fuji H, et al (1993) Brief myocardial ischemia affects free radical generating and scavenging systems in dogs. Heart Vessels 8: 115–120. 8407720 10.1007/BF01744795

[34] Stubbs M, Griffiths JR (2009) The altered metabolism of tumors: HIF-1 and its role in the Warburg effect. Adv Enzyme Regul 50: 44–55. 19896967 10.1016/j.advenzreg.2009.10.027

[35] Singh N, Sharma G, Mishra V, Raghubir R (2012) Hypoxia Inducible Factor-1: Its Potential Role In Cerebral Ischemia. Cell Mol Neurobiol. 10.1007/s10571-012-9803-9 PMC11498632 22297543

[36] Nabetani M, Okada Y, Takata T, Takada S, Nakamura H (1997) Neural activity and intracellular Ca2+ mobilization in the CA1 area of hippocampal slices from immature and mature rats during ischemia or glucose deprivation. Brain Res 769: 158–162. 9374284 10.1016/s0006-8993(97)00819-6

[37] Somjen GG, Aitken PG, Czeh G, Jing J, Young JN (1993) Cellular physiology of hypoxia of the mammalian central nervous system. Res Publ Assoc Res Nerv Ment Dis 71: 51–65. 8380239

[38] Kim JH, Kim KH, Kwon TH, Park YK (2007) Depletion of ATP and release of presynaptic inhibition in the CA1 region of hippocampal slices during hypoglycemic hypoxia. Neurosci Lett 411: 56–60. 17095154 10.1016/j.neulet.2006.10.004

[39] Kim JH, Park YK, Kwon TH, Chung HS (2006) Transient recovery of synaptic transmission is related to rapid energy depletion during hypoxia. Neurosci Lett 400: 1–6. 16644112 10.1016/j.neulet.2006.01.035

[40] Hoye AT, Davoren JE, Wipf P, Fink MP, Kagan VE (2008) Targeting mitochondria. Acc Chem Res 41: 87–97. 18193822 10.1021/ar700135m

[41] Mattiasson G, Shamloo M, Gido G, Mathi K, Tomasevic G, et al (2003) Uncoupling protein-2 prevents neuronal death and diminishes brain dysfunction after stroke and brain trauma. Nat Med 9: 1062–1068. 12858170 10.1038/nm903

[42] Brand MD, Buckingham JA, Esteves TC, Green K, Lambert AJ, et al.. (2004) Mitochondrial superoxide and aging: uncoupling-protein activity and superoxide production. Biochem Soc Symp: 203–213. 10.1042/bss0710203 15777023

[43] Korde AS, Pettigrew LC, Craddock SD, Maragos WF (2005) The mitochondrial uncoupler 2,4-dinitrophenol attenuates tissue damage and improves mitochondrial homeostasis following transient focal cerebral ischemia. J Neurochem 94: 1676–1684. 16045446 10.1111/j.1471-4159.2005.03328.x

[44] Ames BN (2004) Delaying the mitochondrial decay of aging. Ann N Y Acad Sci 1019: 406–411. 15247055 10.1196/annals.1297.073

[45] Ames BN (2004) A role for supplements in optimizing health: the metabolic tune-up. Arch Biochem Biophys 423: 227–234. 14989256 10.1016/j.abb.2003.11.002

